# Multidisciplinary approach in the treatment of tendinous foot involvement in rheumatoid arthritis

**DOI:** 10.1007/s10067-021-05848-8

**Published:** 2021-07-06

**Authors:** Pilar Macarrón Pérez, María del Rosario Morales Lozano, Cristina Vadillo Font, Lidia Abásolo Alcázar, Carmen Martínez Rincón, Benjamin Fernández Gutiérrez, Margarita Blanco Hontiyuelo, María Luz González-Fernández

**Affiliations:** 1 Rheumatology Department, Hospital Clínico San Carlos, Madrid, Spain; 2 Faculty of the E.F.Podiatry, Universidad Complutense de Madrid, Madrid, Spain

**Keywords:** Orthotic-podiatric treatment, Posterior tibial tendinitis, Rheumatic foot, Ultrasonography

## Abstract

**Introduction:**

Patients with rheumatoid arthritis frequently consult for pain resulting from involvement of the tendons of the foot. This pain negatively affects foot biomechanics and quality of life. The most widely used treatment option for this condition is ultrasound-guided steroid injection, while other treatments were recommended such as heel pad, splints, and footwear.

**Objective:**

To evaluate a joint intervention (rheumatology and podiatry) comprising an orthotic-podiatric treatment and infiltrations. We evaluated the response using ultrasound monitoring, a pain scale, functional tests, and assessment of patient satisfaction.

**Methods:**

We performed a non-controlled blinded prospective interventional study of 96 patients with foot pain and selected those with ultrasound-confirmed tendon involvement. Patients enrolled started intervention treatment and were followed for 6 months. The outcome of the intervention was compared with the patient’s baseline status. The pre-post differences in the secondary variables (pain, disability) were analyzed using the t test and contingency tables or the Mann–Whitney test.

**Results:**

Using our protocol, we recorded a rapid and significant reduction in the intensity of pain, in the foot function index, and in the ultrasound parameters (grayscale and Doppler). Structural damage to the tendon improved more slowly, with significant outcomes only at the last visit with respect to baseline. Abnormal foot support was detected in 50% of patients, and 79.5% were using inappropriate footwear.

**Conclusions:**

Our multidisciplinary therapeutic protocol enabled a very significant improvement in tendon involvement. It was well-tolerated, with a high degree of satisfaction, and was easily evaluated using ultrasound. No changes in background medication were necessary.

**Table:** 

**Key Points** • *Multidisciplinary evaluation of patients with RA is advisable because it improves the treatment management in cases of inflammatory activity and structural abnormalities of the foot.* • *Comprising orthopedic-podiatric treatment (heel, splints, and suitable footwear) and infiltrations, in terms of clinical, ultrasound, and functional recovery of the foot tendons.* • *The therapy protocol we propose led to a significant improvement in pain relief and functional recovery.*

## Introduction

Foot involvement is twice as common in patients with rheumatoid arthritis (RA) as in the general population. Approximately 16–19% of patients experience discomfort in their feet at the onset of the disease [1], and up to 90–100% are affected at 10 years [2, 3]. Even in the case of patients whose disease is in remission, residual activity can be observed in the joints of the foot/ankle [1, 4].

The tendons of the foot are very often affected during the initial stages of RA [5, 6]. While any tendon can be affected, those in the medial compartment are most commonly involved, followed by the lateral and posterior tendons. Inflammation of a tendon can affect the structure and biomechanics of the foot [7–9] and is one of the main causes of pain. Control of the condition is challenging because of its high prevalence and negative impact on physical functioning and quality of life [10, 11].

Tenosynovitis is considered to be an expression of the inflammatory activity of RA, and new, specific criteria have been developed to quantify lesions affecting tendons in ultrasound images. Early identification of tenosynovitis, together with treatment, can help to prevent and slow structural damage to the tendon [12].

The most widespread options for treatment of tenosynovitis include steroid injections into the tendon sheath. Ultrasound-guided infiltrations have proven to be more successful than the conventional blind injections. Ultrasound has provided valuable information for the physical examination and has been proven to be highly sensitive for the detection of joint inflammation, tendon involvement, and follow-up [13–15]. Approximately 75% of all infiltrations into a tendon are made in the foot or ankle [16], with short-term relief of pain and of inflammatory symptoms (4–12 weeks) [14, 17]. The technique is simple and well-tolerated and can be performed in the office with minimum complications [18].

Conditions affecting the foot can benefit from other, non-pharmacological interventions, which are recommended by most guidelines on foot care. The most common are foot orthoses, reduction of biomechanical impact, therapeutic footwear, and patient education/self-management [19–22].

The synergy between pharmacological and non-pharmacological options is beneficial for the patient. Multidisciplinary evaluation of the affected foot facilitates management of the disease, thus minimizing its functional impact [23], providing greater satisfaction for the patient, and helping to significantly reduce foot pain and disability [24, 25].

Various guidelines support a multidisciplinary approach to this condition in order to minimize its functional impact [23]. There are also publications that point to the need to evaluate whether non-pharmacological indications, such as functional bandages and exercises to mobilize soft tissues, provide long-term relief of pain [11]. Therefore, the objective of the present study was to carry out a joint intervention (rheumatology and podiatry) comprising orthotic-podiatric treatment and infiltrations for management of tendon involvement in the feet of patients with RA. We also evaluated the response to therapy using ultrasound (OMERACT ultrasound scales) and functional tests.

## Material and methods

### Setting, study design, and population

We performed a prospective, interventional, non-controlled study of patients with RA and involvement of the tendons in the medial and lateral compartments of the ankle diagnosed by ultrasound. Patients were followed up for 6 months. The study was performed at Hospital Clínico San Carlos (HCSC), Madrid, Spain.

Patients were recruited consecutively over 1 year (January 2018 to January 2019) from the Ultrasound Podiatry Clinic of the Rheumatology Department for evaluation of pain in the foot and ankle.

Inclusion criteria are as follows: (a) diagnosis of RA according to the 2010 criteria of the American College of Rheumatology [26], with foot and/or ankle pain; (b) ultrasound-based diagnosis of tendon involvement: posterior tibial tendon (PTT), flexor digitorum longus (FDL), flexor hallucis longus (FHL), and/or peroneal tendons; (c) acceptance of the study conditions; (d) signed informed consent. Exclusion criteria are as follows: (a) age < 18 years, (b) previous history of foot surgery, (c) only active joint involvement and/or involvement of tendons other than those under study (i.e., Achilles tendon and/or anterior compartment tendons).

The study was performed by the same team of professionals from the Ultrasound Podiatry Clinic of the Rheumatology Department (2 rheumatologists and 2 podiatrists). The members of the team had broad clinical experience in the field. This study was approved by the Medical Ethics Committee of the HCSC and was conducted in full accordance with the Declaration of Helsinki (1964). The study comprised 4 visits. At the baseline visit, we recruited the patients and collected a series of variables: demographic data such as height, weight, comorbid conditions, clinical data related to the disease (duration, treatment, activity [28-joint Disease Activity Score or DAS 28]) [27], and disability, as measured by the Health Assessment Questionnaire (HAQ) [28]. We also collected ultrasound variables, namely, degree of inflammatory activity and structural damage, foot pain (quantified using a visual analog scale [VAS] of 0–10) [29], and foot function (using the foot function index [FFI]) [8]. All RA patients underwent a comprehensive podiatric examination by a highly experienced Doctor of Podiatric Medicine (DPM), who was blind to the ultrasound results and to the other clinical findings. At the following visits at 6, 12, and 24 weeks (visits 1, 2, and 3, respectively), a rheumatologist (blind to previous results) repeated the ultrasound examination to evaluate structural damage and inflammatory activity. The grade of pain (VAS) and the FFI were also measured. A patient satisfaction questionnaire was administered at the last visit. The treatment patients took at baseline visit (DMARDs, biologic therapy or both, current corticosteroids use) remained unchanged throughout the study.

### Intervention protocol

All patients with a Doppler signal on ultrasound underwent an infiltration into the tendon sheath with 1 vial of Celestone Chronodose® plus 2 ml of mepivacaine 2%. The infiltration was not performed in the case of patients with partial tendon tear. All patients underwent orthotic-podiatric treatment, which consisted of the following: (a) unloading of the affected tendon using functional splints with supination support straps (medial tendons) or pronating support straps (lateral tendons) for 6 weeks. This could be extended for a further 6 weeks if no improvement was detected on ultrasound; (b) bilateral heel pads [22] (1 cm, ethyl vinyl acetate, 60° shore). In the case of intense involvement (grade 3 tenosynovitis on gray scale or Doppler), the tendon was unloaded initially with a short walker boot for 6 weeks, followed by splints (see above) for at least an additional 6 weeks. In order to guarantee the homogeneity of the orthotic-podiatric treatment, patients received all of their material at the baseline visit. All patients were instructed to perform therapeutic exercises after the second visit and given advice on appropriate footwear.

### Baseline ultrasound assessment

All patients underwent a comprehensive ultrasound assessment, which was performed by a rheumatologist experienced in musculoskeletal ultrasound. This assessment consisted of a systematic longitudinal and transverse multiplanar examination of both feet in exact keeping with standardized scanning techniques [30, 31] in B-mode and power Doppler (PD) mode using a real-time scanner (Mylab 70 XVG, Esaote, Genoa, Italy) equipped with a multifrequency linear probe (10–18 MHz). B-mode and PD machine settings were optimized before the study and standardized for the whole study. These settings were as follows: B-mode frequency, 10–18 MHz; B-mode gain, 56–62%; Doppler frequency, 6.3–14.3 MHz; Doppler gain, 45–62%; low-wall filters; and pulse repetition frequency, 500–750 Hz, depending on the depth of the anatomic area. Bilateral ankle ultrasound examinations were performed using the scanning technique proposed in the latest EULAR standardized procedures [32]. The tendons assessed for the presence of B-mode tenosynovitis, Doppler tenosynovitis, and tendon damage were the peroneus longus and brevis, PTT, FDL, and FHL (a total of 10 tendons in both ankles). All ultrasound findings were documented in at least 2 perpendicular planes. PD examinations were performed according to the indications provided by Torp-Pedersen et al. [33].

B-mode tenosynovitis was defined as abnormal anechoic and/or hypoechoic (relative to tendon fibers) tendon sheath widening, which may be related to the presence of abnormal tenosynovial fluid and/or hypertrophy [34]. A 4-grade semiquantitative scoring system (i.e., grade 0, normal; grade 1, minimal; grade 2, moderate; grade 3, severe) was used to score tenosynovitis detected on grayscale ultrasound [35]. Doppler tenosynovitis was defined as the presence of a peritendinous PD signal within the synovial sheath, seen in 2 perpendicular planes, excluding normal nutrient vessels in the mesotenon and vincula, only if the tendon showed peritendinous synovial sheath widening in B-mode [36]. A 4-grade semiquantitative scoring system (i.e., grade 0, normal; grade 1, minimal; grade 2, moderate; grade 3, severe) was used to score tenosynovitis revealed on Doppler ultrasound. Tendon damage was defined as an internal and/or peripheral absence of tendon fibers or as a complete interruption of the tendon fibers seen in 2 perpendicular planes. A 3-grade semiquantitative scoring system (i.e., grade 0, normal; grade 1, lesion or partial tear; grade 2, lesion or complete rupture) was used to score tendon damage [35].

Pain was evaluated at each visit [29]. Foot function was evaluated using the FFI, which measures pain and mobility limitations that result in foot dysfunction. The scale consists of 23 items divided into 3 subscales: pain (9 items), physical functioning (9 items), and limitation (5 items). Each question was scored using a VAS (0 to 9). The index was calculated by summing the total score for each patient and dividing it by the possible (23 × 9 = 207) and multiplying by 100. Higher FFI scores indicate reduced foot function [8]. The patient’s degree of satisfaction was measured using a 3-question survey with categorical responses.

### Outcome variables

The efficacy of the intervention was based on the improvement in the outcome measures during the follow-up visits compared with baseline. Main outcomes were defined as the improved grade of tenosynovitis on the ultrasound scales, inflammatory activity (Doppler), and structural damage to tendons. Improvement was defined as a reduction of at least 1 grade in the category of inflammation and/or Doppler signal and/or structural damage. Secondary outcomes are as follows: as (1) improvement in the intensity of pain, (2) improvement in the FFI, (3) patient satisfaction. Other variables included sex; age; body mass index (BMI); time with RA; HAQ; DAS28; employment; participation in sports; smoking habit; and comorbidities: arterial hypertension (AHT), diabetes Mellitus (DM), heart disease (coronary artery disease), osteoporosis, depression. Current treatment (DMARDs, biologic therapy, or both) and current corticosteroids use remained unchanged throughout the study. Footprint type, type of structural foot pathology, steroid injection number.

### Statistical analysis

Accepting an alpha risk of 0.05 and a beta risk of 0.2 in a bilateral contrast, 50 tendons are required to detect a difference equal to or greater than 25%. The percentage of follow-up losses has been estimated at 35%. Sociodemographic and clinical characteristics were reported as a frequency distribution and the mean and standard deviation or median and interquartile range. We analyzed the differences between the baseline visit and visits 1, 2, and 3 with respect to the degree of ultrasound involvement: improvement and/or worsening, FFI, and pain (VAS). The Mc Nemar test was used to analyze the result of the pre-post intervention for the main variables; factors associated with the response to treatment were analyzed using regression analysis. Differences in the secondary variables (pain, disability) were analyzed using the *t* test and contingency tables or the Mann–Whitney test depending on the sample size. All analyses were performed using Stata v.13 (Stata Corp., College Station, TX, USA). A 2-tailed *p* value under 0.05 was considered to indicate statistical significance.

## Results

**Fig. 1:**
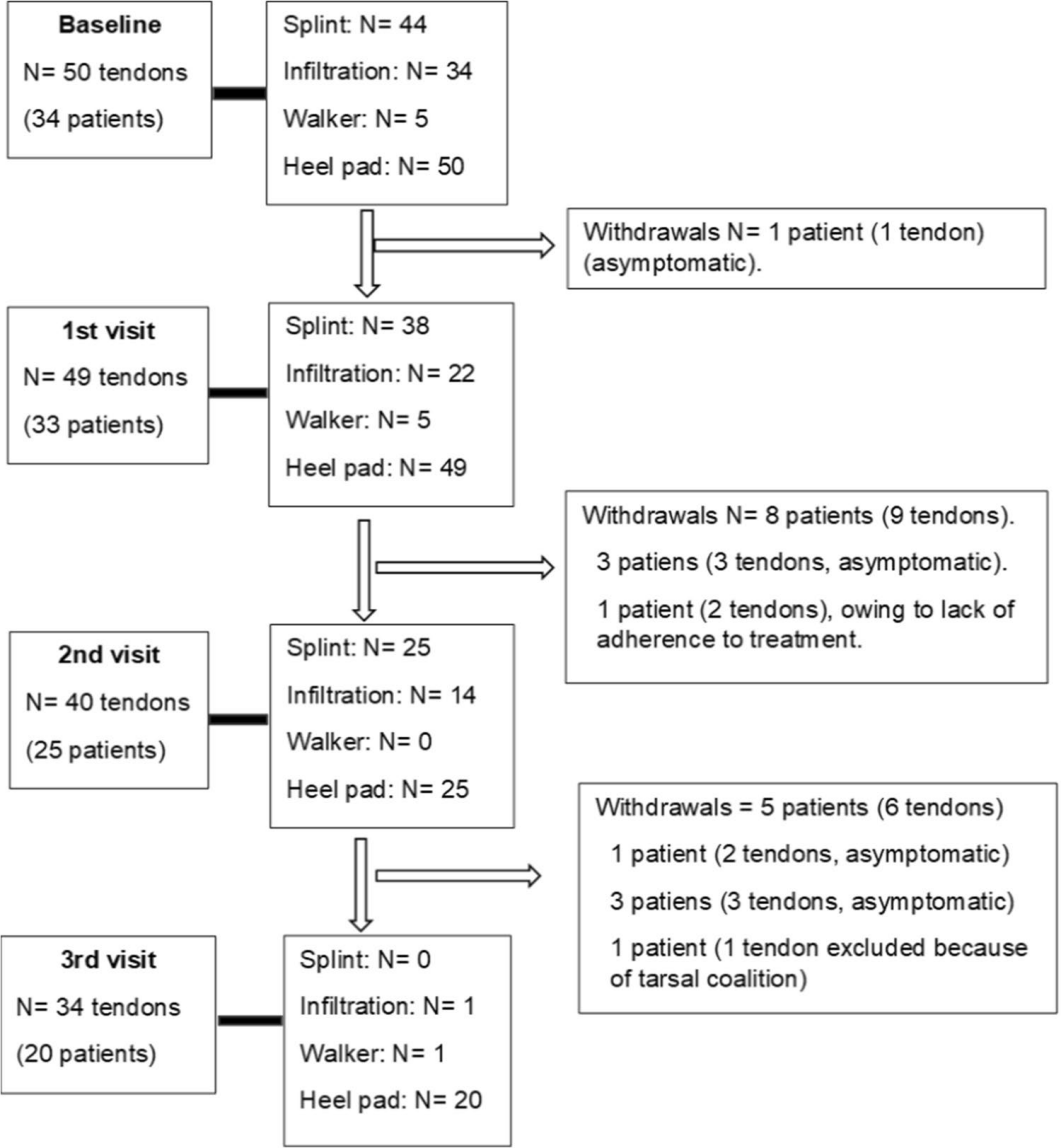
Flowchart showing patients’ progress through the study

We preselected 96 consecutive patients sent for ankle and foot pain. Of these, we selected 34 meeting the inclusion criteria, who agreed to participate in the study. As involvement was bilateral in 16 patients, we treated a total of 50 tendons. The flow of patients through the study is shown in Fig. 1.

The baseline characteristics of the patients are shown in Table 1. There were 29 women (85.3%), and the mean age was 55.9 (46.7–60.8) years. Median (IQR) time with RA was 10 (6–20) years. Disease was moderately active (median DAS28, 3.95 [2.86–5.53]; median HAQ, 0.87 [0.4–2.1]; median CRP, 4.18 [2.86–5.53]). A total of 27 patients (50%) were overweight (BMI, 25–29.9), 13 (29.4%) were obese (BMI, 30–39.9), and 7 (20.6%) had a normal (BMI 20–24.9). Only 5 patients (15%) exercised regularly. A total of 26 patients (76.5%) worked outside home. Ten patients (29.4%) were on sick leave, and 13 (38.2%) had been smokers, 7 (20.6%) smoked during the study. Comorbidity was present in 33 patients (76.7%), as follows: arterial hypertension, 10 (29.4%); diabetes mellitus, 3 (8.8%); heart disease (coronary artery disease), 2 (5.8%); osteoporosis, 13 (38.2%); and depression, 5 (14.7%).

**Table 1:** Baseline characteristics of the 54 patients

Variables	Value
Female sex, n (%)	29 (85.3)
Age (years), median (IQR)	55.9 (46.7–60.8)
Time with RA (years), median (IQR)	10 (6–20)
HAQ, median (IQR)	0.87 (0.4–2.1)
DAS28, median (IQR)	3.95 (2.86–5.53)
Positive ACPA titer, n (%)	15 (46.9)
Positive RF, n (%)	17 (53.1)
Pain present, n (%)	34 (100)
Median (IQR) pain VAS	8 (7–9)
Median (IQR) FFI	55.9 (38.1–66.2)
Tendons affected, n (%)	50 100%
Posterior tibial	35 (70)
Short peroneal tendon	4 (8)
Long peroneal tendon	2 (4)
Flexor hallucis longus	9 (18)
Flexor digitorum longus	0
Footwear, n (%)	
Appropriate	7 (20.6)
Inappropriate	27 (79.4)
Footprint, n (%)	
Flat or severe cavus (grades 3 and 4)	14 (28.0)
Normal or flat or cavus (grades 1–2)	36 (72.0)
Type of foot, n (%)	
Neutral (normal)	25
Flat	14
Pronated	9
Supinated	2

The treatment administered is shown in Table 2. The number of infiltrations decreased from 34 at baseline to 1 at the final visit. Use of splints decreased from 44 to 0, in parallel with the improvement in clinical and ultrasound findings. The baseline ultrasound scan (grayscale) showed that 72% of the tendons were affected by grade 2 or 3 tenosynovitis and 22% were affected by grade 1 disease. In the last visit, grade 1 tenosynovitis persisted in only 29%, and the remainder were normal (p < 0.000). A Doppler signal was observed at baseline in 68% (24%, 38%, and 6% for grades 1, 2, and 3, respectively) and at the final visit in only 17.2% (p < 0.000) (Table 3). Patients who did not attend the visits were followed by telephone to determine the reason for not attending: 13 patients (16 tendons) decided not to continue because they were asymptomatic and, as they were active workers, they had problems to attend the appointments at the outpatient clinic.

**Table 2:** Treatment of tendons at baseline and follow-up visits (N = 50 tendons [%]). Heel pads were maintained bilaterally in all patients who attended the visits

	Baseline n (%)	Visit 1 n (%)	Visit 2 n (%)	Visit 3 n (%)
Splint n (%)	44 (88)	38 (76)	25 (62.5)	0
Mean (SD) weeks using the splint	13 (6.5)			
Median (IQR)	12 (6–18)			
Infiltration, n (%)	34 (68)	22 (44)	14 (35)	1 (3.03)
Median (IQR) no. of infiltrations/tendon	2 (1–2)			
Mean (SD)	1.7 (0.9)			
Number of infiltrations per tendon, n (%)				
0	17 (34)			
1	8 (16)			
2	17 (34)			
3	8 (16)			
Walker boot, n (%)	5 (10)	5 (10)	0	1 (3.03)

**Table 3:** Results of the different values in successive visits

	Baseline visit N** = **50	Visit 1 N** = **49	Visit 2 N** = **40	Visit 3 N** = **34
Foot index				
Median (IQR)	56.34 (39–67)	29.30 (21–38)	21.57 (12–29)	11.5 (2–13)
Median difference with baseline		− 22 (− 39, − 6.1)	− 35 (− 47, − 13)	− 46 (− 56.34)
p value		p** = **0.0000	p** = **0.0000	p** = **0.0000
Problem resolved (%)				
Not at all		5 (12%)		0 (0%)
Some		10 (20%)		4 (11.4%)
High		26 (52%)		19 (54.3%)
Completely		8 (16%)		11 (31.4%)
US (grayscale) (%)				
Grade 0	3 (6)	22 (44.9)	17 (42.5)	24 (70.6)
Grade 1	11 (22)	16 (32.6)	16 (40.4)	10 (29.4)
Grade 2	26 (52)	7 (14.3)	7 (17.5)	0
Grade 3	10 (20)	4 (8.1)	0	0
p value		p** = **0.000	p** = **0.000	p** = **0.000
US (Doppler) (%)				
Grade 0	16 (32)	30 (61.22)	25 (62.5)	28 (82.3)
Grade 1	12 (24)	11 (22.45)	8 (20)	3 (8.8)
Grade 2	19 (38)	7 (14.3)	7 (17.5)	3 (8.8)
Grade 3	3 (6)	1 (2)	0	0
p value		p** = **0.009	p** = **0.0017	p** = **0.0000
Structural abnormality (%)				
No	17 (34)	22 (44.9)	17 (42.5)	29 (85.3)
Yes	33 (66)	27 (55.1)	23 (57.5)	4 (5.6)
Rupture	0	0	0	1 (2.8)
p value		p** = **0.307	p** = **0.4112	p** = **0.0000

### Efficacy

**Fig. 2:**
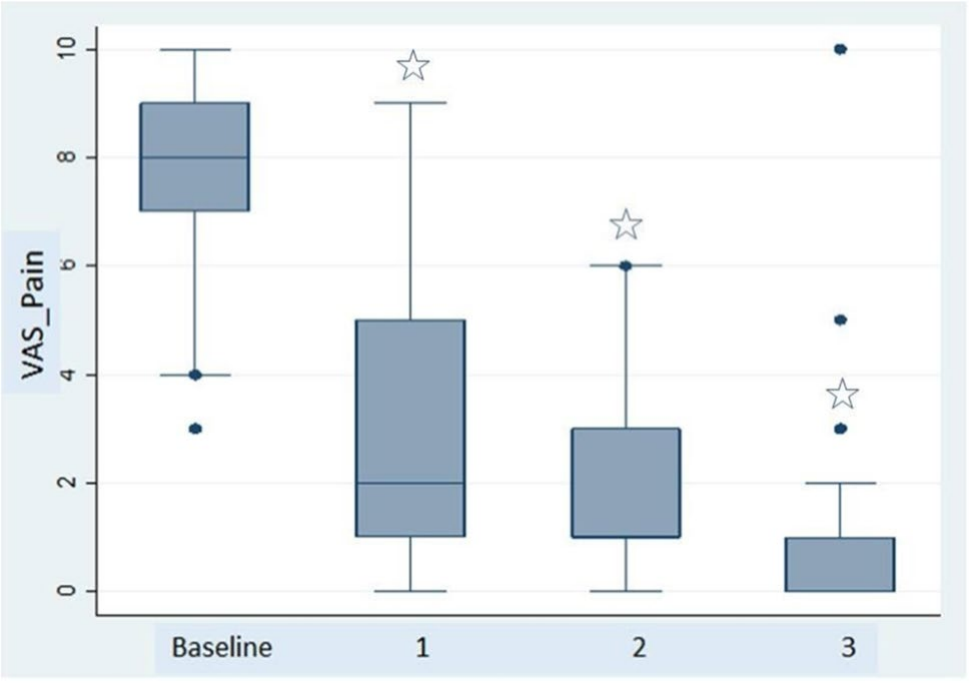
Progress of pain over the follow-up visits measured using a visual analog scale (0–100). The boxes show the median (IQR). The comparisons were made between the baseline visits and the individual follow-up visits (*p = 0.000)

**Fig. 3:**
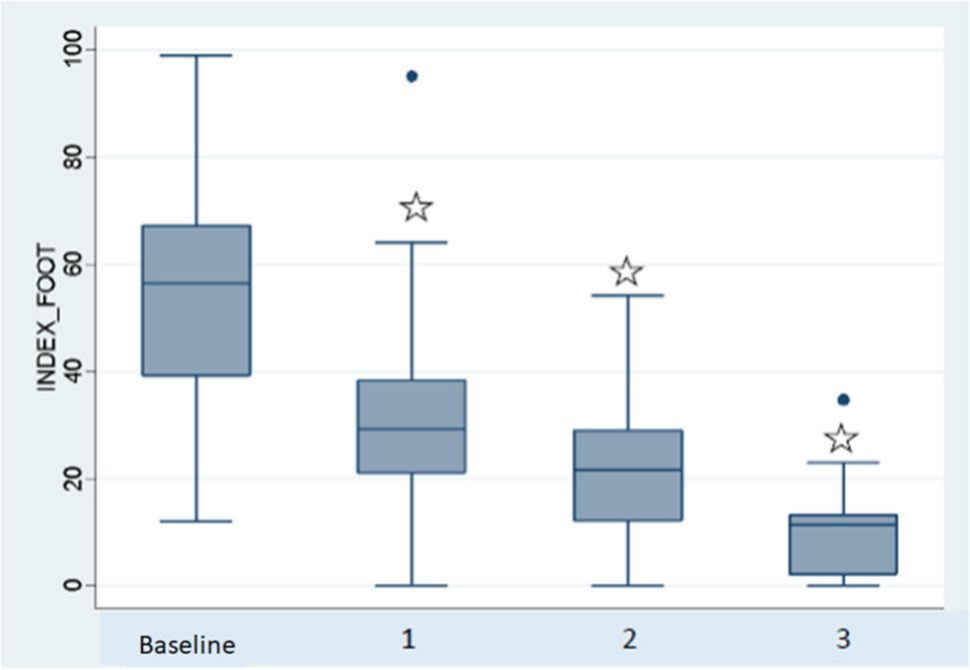
Progress of foot function index over the follow-up visits measured using a visual analog scale (0–100). The boxes show the median (IQR). The comparisons were made between the baseline visits and the individual follow-up visits (*p = 0.000)

The baseline values and changes in the outcome measures during the visits are shown in Table 3 shows. All of the parameters assessed during the study improved rapidly and progressively; the improvement was statistically significant at each visit compared with baseline. Pain decreased in intensity rapidly and strikingly from a baseline median score of 8 (7–9) to 2 (1–5) at visit 1, then to 1 (1–3) at visit 2, and to 0 (0–1) at the final visit (p = 0.0000, all comparisons). At the end of the study, the intensity of the pain (VAS) was 0 in 52% and ≤ 1 in 48% of the tendons (Fig. 2). The FFI (0–100) improved progressively, falling from 56.34 at baseline to 29.56, 20.15, and 12.00 at visits 1, 2, and 3, respectively (p = 0.0000, all comparisons) (Fig. 3).

### Ultrasound parameters

Improvement in grayscale ultrasound tenosynovitis was significant from visit 1 and continued at the subsequent visits until the end of the study (p < 0.000, all comparisons). The frequency of grade 2 and 3 tenosynovitis decreased throughout the study (72% at baseline vs 0% at the last visit). Doppler signal ultrasound in tenosynovitis: The decrease in the Doppler signal with respect to baseline was significant (p = 0.000) from visit 1 and persisted in visits 2 and 3. Structural alteration of the tendon was evaluated in terms of presence/absence of partial intratendinous tear. No structural alterations were observed in 34% of tendons at baseline; this percentage increased to 85% at the end of the study. There was only 1 case of subtotal tear of a PTT, which occurred between visits 2 and 3. The structural damage to the tendon improved more slowly than the other ultrasound abnormalities (tenosynovitis and Doppler signal); the difference was only statistically significant between baseline and visit 3 (p = 0.000).

### Patient satisfaction

The treatment protocol was well-received. All patients were satisfied with the program (100%). Patients considered their condition to have improved “considerably or completely” in 85.7% of cases. According to 85% of the patients, the program had “considerably or completely” resolved their foot problem at the end of the study.

## Discussion

The combination treatment protocol used in this study proved to be highly effective in terms of clinical, ultrasound, and functional recovery of the tendons of the foot. The therapeutic methods applied were well-accepted and well-tolerated and highly valued. Patient satisfaction was better than reported in other rheumatology departments with multidisciplinary podiatry teams where prevention was based on education and footwear [24, 28].

We found that 35.41% of patients with RA referred for foot pain had tenosynovitis, frequently affecting both feet (47.06%). In contrast with other authors [5, 6], tendinopathy was not often observed in the initial phases of the disease, since the median course of RA was 10 years. Therefore, it is important to remember that tendons may be affected at any point during the course of the disease. As reported elsewhere [5, 7–9], we also observed a greater prevalence of PTT involvement (70%), followed by the peroneal tendons. In one study [37], the authors suggested that evaluation of the PTT should be part of the evaluation of structural damage in patients with RA. We observed an alteration in the support and structure of the foot in 50% of cases. In 92%, the feet were flat or pronated, and in the remaining 8%, they were cavus or supinated. Footprint morphology was severely affected by pronation or flattening in 29.4% of patients and mildly affected in 20.6%. This finding can be explained by the association between PTT involvement and flat/pronated foot [38, 39], since it was the most affected tendon in our sample.

As reported by several authors [13, 15, 17, 18, 40], ultrasound-guided infiltrations improve the symptoms of tenosynovitis, irrespective of the site. However, the maximun efficacy occurs only during short term (1–3 months) [14]. Considering these published studies as the usual clinical practice, the novelty of our study lies in the use of splints to relax the tendons, since the foot is subject to considerable loading, both during movement and when static. Reducing stress on the tendons is particularly important in patients with structural abnormalities of the foot. Known factors that negatively affect foot involvement in RA are obesity [41, 42] and inappropriate footwear [43, 44]. We found that 79.4% of patients used inappropriate footwear and that the same proportion was obese or overweight. Hence, we believe that it is very important to provide patients affected by rheumatic diseases with information on diet and footwear as soon as their disease is diagnosed, especially if their occupational activity involves prolonged standing or if they regularly play sports. Pain had a negative effect on quality of life and functional disability [10, 11]. We managed to reduce pain rapidly and significantly, thus improving quality of life. This improvement was in parallel to that of the FFI. We found no publications that performed a detailed follow-up of pain and the FFI in tendon involvement with respect to clinical and ultrasound improvement. Ultrasound variables improved significantly. The ultrasound scales are easy to use and have proven very useful in the follow-up of tendon involvement, since they help when choosing therapy [40].

Some publications indicate the need to evaluate whether non-pharmacological interventions (functional bandages and mobilization exercises) provide long-term relief [11]. Therefore, we designed this study, in which tendon involvement in patients with RA was evaluated and treated jointly by a rheumatologist and a podiatrist. Treatment was monitored using ultrasound based on the scales of OMERACT.

Our study is subject to a series of limitations: (a) there was no control group treated only with infiltrations; nevertheless, with the design used, we were able to show the efficacy of intervention. Furthermore, taking into account that our study measures efficacy at 3 and 6 months and that an infiltration lasts an average of 3 months, in the last visit, we would not have the effect of the infiltration. (b) Some patients did not adhere to treatment. (c) Several patients did not attend the follow-up visits as they were symptomatic.

Multidisciplinary evaluation of patients with RA is advisable because it improves the treatment management in cases of inflammatory activity and structural abnormalities of the foot. Involvement of foot tendons in RA leads to disability, functional limitation, and pain that can be as severe as that of joint pain. However, the indexes used to measure disease activity do not include the evaluation of the tendons in patients with RA. Therefore, we believe that assessment of the patient with RA should include not only joint involvement, but also an examination of the affected tendons. The therapy protocol we propose led to a significant improvement in the study variables, namely, relief of pain and improved functioning. The protocol was well-tolerated and easily evaluable by ultrasound, with no need for changes in the patient’s background medication. Therefore, we believe that it should be advisable to incorporate it into daily clinical practice.

In case of not having ultrasound equipment, doctors can request the ultrasound examination to the central radiodiagnosis services.
